# Head and Neck Surgery Residency During Covid-19 Pandemic. Lessons from Southern Italy

**DOI:** 10.37825/2239-9747.1008

**Published:** 2020-10-01

**Authors:** P De Luca

**Affiliations:** 1 Department of Medicine, Surgery and Dentistry, University of Salerno, Salerno, Italy

Dear Editor,

On February 2020, the first case of a patient affected by SARS-coronavirus-2 (SARS-CoV-2) was confirmed in Codogno (Lombardia, Italy); this date will be remembered as the beginning of the coronavirus disease-19 (COVID-19) pandemic outbreak in Italy^1^.

At the peak in mid-April, there were more than 2 million cases of confirmed COVID-19 globally, and more than 150.000 deaths; since the spread of the SARS-CoV-2, Italy has been one of the most affected countries in the world and one with the highest rate of COVID-19 related deaths^2^.

From March 10, the beginning of the lockdown in Italy, all academic medical institutions have begun to face a unique set of challenges to ensure the safety of resident physicians and the stability of the residency programs^3,4^.

I am an otolaryngology-head and neck surgery resident physician in Salerno, Italy.

From the beginning of the pandemic, me and my residency colleagues have been actively employed in dealing with the emergency, although fear and uncertainty were the predominant feelings, both for us and for our family. The emergency unit of nasopharyngeal swabs was entrusted to otolaryngology residents, who are turning for months to ensure this service to the Hospital; as suggested by the most recent scientific literature, we performed nasopharyngeal swab and not combined nasal/throat swab^5^

Moreover, we guaranteed the outpatient clinic for urgent visits, and the shift in the operating room.

This unprecedented situation required a longer period of mechanical ventilation in COVID-19 critically ill subjects, so these patients has been considered for tracheostomy: the use of tracheostomy can potentially increase the availability of intense care unit beds but provided an unique challenge for head&neck surgeons and for healthcare workers. During our rotation, we assisted our consultants during tracheostomy; the procedures were performed by expert surgeons to guarantee fast and effective tracheostomy and to avoid long infection exposure.

In this scenario, we are facing a double challenge: on one hand, we are forging our character and improving our skills during this pandemic. On the other hand, diagnostic and therapeutic procedures were limited to emergencies and oncology patients^6^, with a substantial decrease in the residents’ involvement in the surgical theater, so we are not able to quantify the short-term and the long-term implications of these changes.

In the absence/reduction of hands-on surgical experience, we should consider newly available options for trainees, also considering the need to respect social distancing.

While we understand the importance of in-person patient experience, we should consider the opportunity of virtual education and telemedicine; due to the impossibility to participate in lectures, we strongly believe that departments should organize online lessons and virtual academic conferences^7^. In this view, a simple idea could be to develop teaching contexts in telemedicine, dealing contracts with companies that provide online courses and distance learning. We also recommend viewing high-quality surgical videos to help make up for the significant loss of time in the operating room.

In addition, to bridge the surgical gap caused by the COVID-19 pandemic and to not limit surgical training to oncological patients and trauma emergencies, I suggest a greater involvement of residents in the operating room.

I would also like to emphasize the importance, for a University with surgical residencies, the presence of a permanent anatomical dissection laboratory, which allows residents to improve their surgical skills and to learn new techniques; the dissection time can be also considered as a moment of sharing between residents and tutors6.

Although no one can now quantify how much our surgical education will be compromised by the COVID-19 pandemic, I believe that this unprecedented circumstance will change the way we will be educated. I am optimistic that future otolaryngology residents will benefit from these changes, and I am sure that those of us training during this pandemic are deeply changed and grow in our role as physicians and future leaders.
